# MLuq Protocol: A Proposal for the Immobilization of the White Weapon, Preservation of DNA Traces, and Its Chain of Custody

**DOI:** 10.3390/healthcare11111573

**Published:** 2023-05-27

**Authors:** Manuel Luque-Oliveros, Salvador Martínez-Flores, Rubén Morilla-Romero-de-la-Osa

**Affiliations:** 1 Servicio de Cirugía Cardiovascular y Área del Corazón—Cirugía Torácica del Hospital, Universitario Virgen Macarena (HUVM), 41009 Sevilla, Spain; 2 Departamento de Enfermería, Facultad de Enfermería, Fisioterapia y Podología, Universidad de Sevilla, 41009 Sevilla, Spain; 3 Instituto de Biomedicina de Sevilla (Hospital Universitario Virgen del Rocío/CSIC/Universidad de Sevilla), 41013 Seville, Spain; 4 Centro de Investigación Biomédica en Red de Epidemiología y Salud Pública (CIBERESP), Instituto de Salud Carlos III, 28029 Madrid, Spain

**Keywords:** wounds, stab, case management, forensic medicine, DNA contamination

## Abstract

Unprecedentedly, this article presents a useful management protocol for the workers in emergency situations assisting victims of white weapon aggressions with a dual innovation. It could presage a possible advance in the healthcare management of these patients and support important repercussions in the legal field when this type of wound is inflicted due to an aggression. The MLuq protocol has been agreed by consensus in a multidisciplinary manner including experts belonging to the state security forces (judicial and scientific Police), to the healthcare area (surgical nursing, emergency medicine, general cardiothoracic and digestive surgery, and the legal and forensic medicine area), to the legal system (a jurist specialized in the area), and to the academic sphere. It is the first paper to propose purse string sutures as a weapon immobilization technique, as well as a set of actions designed to obtain biological traces of legal interest and to preserve the chain of custody. Therefore, it is a useful tool for the health and legal personnel, and especially for the victims.

## 1. Introduction

Traumatic pathology is a severe public health problem at the global level, responsible for around 14,000 deaths a day [1]. According to data from the National Institute of Statistics, of the 493,776 deaths recorded in 2020 in Spain, 16,078 (3.2%) were due to external causes, with the number aggressions (homicides) among them amounting to 289 (1.8%) [2]. Traumatic pathology is a potential cause of mortality and of sequelae in the survivors—a reason why it is a severe or potentially severe disease. Quantifying the severity of a trauma case is very important for different reasons, namely, to allocate the emergency resources that are most appropriate for severity of the case, and to initiate early treatment and transportation to the most appropriate trauma care hospital center according to severity [1].

Among the variants of traumas, penetrating traumas are the main cause of early death and permanent disability, with sequelae that are not only restricted to the physical plane but also to the emotional responses when traumas occur in a context of aggression [3,4].

In our area, traumas due to white weapons are the most common cause of penetrating wounds [5]; a direct and abrupt application of a mechanical force over a focused area is the mechanism of action, generally through the penetration of objects that pierce the skin and generate an open wound of varying depth [6]. When penetrating traumas reach a blood vessel of an important caliber, they pierce it, and the hemorrhage exposes the subject to an impaired hemodynamic state with possible hypovolemic shock and multiple organ dysfunction with an agonic condition due to the accumulation of blood in the cavities [3].

Statistically, from 55% to 65% of the individuals with fatal traumas due to white weapons die in the prehospitalization stage [7]. It has been shown that the survival probabilities increase as the time between the event and the assistance provided to the patient decreases [8]. Therefore, healthcare management must adhere to protocols guided by standardized criteria [9], whose main intervention is stabilizing the patient [5].

Although this is widely known due to the experience gained in the clinical practice, there is no specific protocol for the stabilization of white weapons, given the diverse evidence available.

On the other hand, the white weapon can be manipulated by different professionals, which is a reason why the DNA or fingerprints of the person brandishing it at the time of aggression (transferred DNA or fingerprints) can be altered [10]. If this should happen, the offender’s genetic identification process [11], and consequently the chain of custody [12] (adequate retrieval of the biological trace, preservation, individualization, appropriate transportation, and controlled delivery), might be severely affected [12,13].

Briefly, the chain of custody is a set of acts whose purpose is the collection, transfer, and conservation of evidence or biological traces obtained in the course of a criminal investigation in order to ensure the authenticity, inalterability, and indemnity of the evidence [13].

Currently, the Courts of Justice deny the legal validity of proofs in proceedings due to contamination or manipulation of the evidence presented. Thus, collection, transportation, and custody of the sample gains relevance, as authenticity, inalterability, and indemnity of the evidence must be ensured [12,13].

Although the competent Investigating Judge demands full guarantee of the sample’s chain of custody [14], an absence of regulation ensuring the chain of custody of DNA evidence is currently perceived in our legal system, which causes legal insecurity and polemical issues [14].

Based on the judicial police, forensic physicians, and accredited laboratories’ praxis, they collect and analyze the conviction pieces that shall constitute proofs through their own performance protocols and on post-mortem victims [15]. However, there are no protocols for taking samples of interest in the case of a patient with a stab wound who is still alive at the crime scene when the emergency services arrive. This fact is both significant and transcendental, as there are still a number of controversies in the performance of healthcare workers in the face of victims of an alleged crime resulting in injuries, as the chain of custody is not entirely ensured [13]. Therefore, the criminal proceeding is oftentimes inadequate, deficient, and declared null due to the evidence presented [16].

To date, the development of work protocols in the clinical assistance area and the forensic-legal area have been developed independently since each area has specific objectives. In this sense, it would be new and useful to draw up action protocols that would be useful for both areas together.

In this context, we set out the objective of establishing a performance protocol for health personnel assisting patients with penetrating traumas by melee weapons which allows for the following: (i) preserving the biological traces present in the handle or retrieving DNA from the melee weapon or from another specific area on the patient during the ensuing healthcare ensuring the immobilization of the stab; (ii) making a contribution to the reliability of the chain of custody and collaborating with the legal authorities handing them in the samples collected.

## 2. Materials and Methods

Design: The current paper follows a theoretical–conceptual design as an initial step for the implementation and evaluation of the protocol. A previous bibliographic review was conducted, following the indications set forth in the PRISMA statement (Spanish version), to verify the non-existence of protocols or recommendations in this regard. For the different search strategies implemented, and flowchart can be seen in the Supplementary Materials.

Application scope: Nurses and physicians developing their care practice in public health emergency companies, the hospital emergency service, extra-hospital medical emergencies, primary care urgency services, and hospital surgical urgency services.

Target population: Any patient/client or user that has been a victim of stabbing with any melee or similar weapon requiring emerging healthcare to save their life, and with the certainty that the weapon was not been later manipulated by any passer-by or other individuals after the stabbing. The victims excluded will be those with a melee weapon wound resulting in death or other stabbed patients who have already died, as a forensic physician and the legal police will be called upon in these cases.

### 2.1. Material Required during the Procedures to Be Performed

- Specific surgical instruments (Adson dissection forceps, no teeth or similar; Crile Wood needle holder or similar; Rochester hemostatic forceps or similar; and Mayo surgical scissors or similar);
- Tourniquet set and Sure-Snare tourniquet kit;
- Surgical suture threads (36″-90 cm and 30″-75 cm)
- Lidocaine hydrochloride 2%, injectable solution or similar;
- 10 mL sterile syringes, 18 G, and 21 G needles;
- Polaroid camera;
- Disposable gloves, sterile gloves, and surgical masks;
- Sterile nylon stable swabs (for samples) (FLOQSwabs, COPAN, Brescia, Italy) and sterile saline serum;
- Packaging cardboard boxes or paper bags (different sizes).

### 2.2. Protocol Elaboration Phases

1. Conceptual phase: Conceptualization of the protocol was the responsibility of the main author (M. Luque), from whom its name is derived (MLuq protocol), with its initial design submitted to review and approval by the other authors in order to ensure its scientific–technical, sanitary, and legal quality. As these three areas are very different, the participation of several collaborators with proven experience in each area was necessary.

2. State-of-the-art assessment: As in any research and innovation process, previous published information was sought in order to learn of antecedents or already established possible solutions to the problem in question. To such an end, a bibliographic review was conducted in the PubMed, Cochrane, LILACS, SciELO, Scopus, Embase, and Trip databases, following the indications set forth in the PRISMA statement (Spanish version), to verify if there are any protocols or recommendations in this regard. A MeSH/DeCS term combination was employed, and the search criteria were focused on the last 5 years (2017–2021) and on articles published in English and in Spanish (see Supplementary Materials).

3. Writing of the protocol: The protocol was written step by step, conceptualizing the chain of actions or procedures to be followed from the moment the health personnel find a stabbing victim, aiming to obtain biological traces of legal interest and contributing to preserving the chain of custody, without neglecting emergency health care or the legal responsibilities related to it.

4. Performance algorithm: Once the entire protocol step has been defined, a performance algorithm was prepared with the intention to easily systematize it, ensuring its reproducibility, and observing the aforementioned standards.

5. Final review and approval of the protocol: Professionals who had more than 10 years of experience in their respective fields and who are currently active were contacted. The protocol and its algorithm were reviewed by experts belonging to the state security forces (judicial and scientific police), to the healthcare area (surgical nursing, emergency medicine, general cardiothoracic and digestive Surgery, and legal and forensic medicine area), to the legal system (a jurist specialized in the area), and to the academic sphere (University of Seville and University of Pablo de Olavide).

Interviews were carried out with all of them, in which a first version of the protocol was presented. They were asked the following questions: (1) Is there any protocol currently in your workplace that allows healthcare for people who have been stabbed, while defining a weapon immobilization strategy while preserving DNA samples from the aggressor and their chain of custody? (2) What technical limitations from your area of knowledge do you perceive in the protocol? So, each professional contributed what he considered appropriate from a health, forensic, or legal point of view. The appropriate changes were implemented, and the final protocol was presented at a multidisciplinary professional conference organized by the first author (MLuq) in our city, where these experts gave their opinion about the final protocol and its usefulness, and the final version of the protocol was approved at the close of these conferences.

6. Registration of intellectual property: Finally, the protocol was registered in the Andalusian Territorial Registry of Intellectual Property with file number RTA-582-22.

### 2.3. Ethical Aspects

Since it is a conceptual study that has not yet been implemented in clinical practice, this study is exempt from evaluation by an ethics committee, from the request for informed consent, and other ethical requirements that biomedical research must submit to. No experts consulted received financial or material gratification. All participated voluntarily and selflessly.

## 3. Results

None of the experts responded positively to the question about the existence of a similar protocol in their area of work and all agreed on the suitability and usefulness of creating one.

The MLuq protocol is a series of concatenated actions designed to stabilize a patient in a given emergency situation, as well as for the effective collection of biological traces of legal interest to contribute to its input in the future investigation phase. In addition, the protocol includes a previous evaluation phase to determine its activation by the health personnel according to the emerging situation in question.

**Figure 1:**
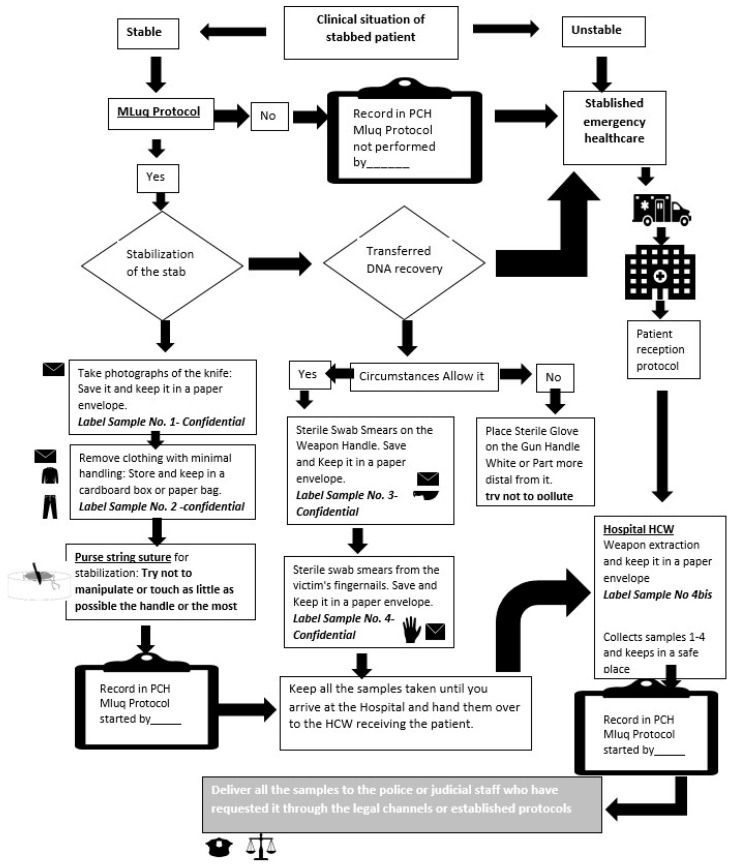
Algorithm of the MLuq protocol.

Each of these phases is briefly described below, in order to show the chain of actions, which are also schematized in the algorithm (Figure 1). The full protocol is attached as complementary material due to the restriction in the number of words associated with the article.

Previous evaluation: Health-related evaluation of the patient according to the mandatory guidelines and care algorithms. Identify the location of the weapon, assessing displacement risk during the assistance provided. Make sure that nobody has touched the handle after penetration into the victim and that the aggression was perpetrated by a third party. In such case, initiate the protocol.

Phase 1., Stabilizing the weapon: Wearing disposable gloves and a mask, take several photographs of the white weapon and of the patient’s position. Put the photographs in a paper envelope labelled as ‘Sample No. 1 (confidential)’. Remove the patient’s clothes and keep them in a cardboard box or paper bag (never plastic, to avoid DNA degradation), trying to manipulate it as little as possible and labeling it with the patient identification data and with the’ Sample No. 2 (confidential)’inscription. Verify that the samples collected are in a safe place with no possibility of exposure to manipulation or contamination. Consciously watch over the samples until they are handed in to the pertinent personnel in due time.

The next step is to stabilize the weapon by applying local anesthesia around the penetration site. Perform the purse string suture starting with the most proximal part, leaving the handle free, avoiding contact and contamination as much as possible. After suturing, apply the following: (i) uncontrolled pressure (in the case of light weapons)—tie both suture threads, applying enough pressure to immobilize the melee weapon together with the patient’s skin; in addition, cut both leftover threads leaving approximately 3 cm in length; or (ii) controlled pressure (in the case of heavy weapons)—cut the suture thread needle leaving the maximum length possible in both distal ends. Bring both leftover suture threads closer and apply the thread guide or tourniquets, as required by the situation. Tauten the most distal leftover thread and bring the thread guide or tourniquet closer to the weapon, exerting a pressure that allows the closure of the circular suture performed.

Place the hemostatic forceps (Rochester or similar), grabbing the suture thread and thread guide or tourniquet at the same time. Verify and watch over stabilization of the weapon. If necessary, repeat the previously described steps.

Phase 2, Preservation of circumstantial evidence and retrieval of transferred DNA: Inform the team about initiation of the phase according to the concrete circumstances of the case so that nobody interferes.

Put on sterile gloves and keep the mask on to avoid possible contamination. Take the sterile nylon swab out of its original packaging, impregnate its cotton part with saline serum and slide it (apply with semi-circular back and forth movements up to five times on the entire surface) over the handle to collect the DNA sample. Slide the impregnated swab with repetitive and semi-circular back and forth movements up to five times on the entire surface of the handle or of the visible or most protruding distal external area of the penetrating object.

Put the swab in the cardboard box or paper bag. Note down the patient identification data on the outer part and write ‘Sample No. 3 (confidential).’ Check proper closure to prevent it from opening and contaminating the sample. In case of swab contamination, open a new one and repeat this action. If the patient presented resistance to the aggressor, take a sample with another swab from the fingernails, between the hyponychium and below the nail plate end. If the circumstances do not allow for retrieving the DNA from the melee weapon, a sterile glove shall be placed on the weapon’s handle so that it is not touched or manipulated until its removal in the surgical area.

Phase 3, Patient transfer and reception at the reference center with the samples under custody: Transfer the patient according to the center’s protocol and record the identification data of the professionals in charge of the delivery–reception of the patient and samples under custody in the medical chart or sheet authorized for such a purpose (note down the date, time, place, and health institution or service where the patient will stay). Hand in a photocopy of the record made during the assistance provided and notes of the samples collected and under custody to the nursing or medical professional of the receiving center that assumes responsibility for the patient. Verify who the person in charge of reception is and that all the documentation received is correct, and consciously store the samples collected in a safe place, avoiding contamination, until they are requested by the pertinent authorities through the established legal channels.

Verify the MLuq protocol annotation in the patient’s medical chart or in the sheet authorized for such purpose. Notify the legal authority of the patient with a penetrating melee weapon at the hospital as soon as possible and according to the center’s own protocol. Once the melee weapon has been removed, wearing sterile gloves and a mask, place the handle without touching it (to the extent possible) in a cardboard box or paper bag. Note down the patient identification data on the outer part of the box containing the melee weapon and write ‘Sample No. 4 bis (confidential)’. Check proper closure to prevent it from opening and contaminating the trace. Fill out the corresponding injury or legal report.

When requested, hand in the documentation referring to the patient along with the samples under custody at a single moment and without mutual interference once they have been accepted and the request has been recorded. Note down the following in the patient’s medical chart or in the sheet authorized for such purpose: identification of the legal or fiscal body, or of the requesting/receiving police officer, with proof of the membership body and plate number; information requested; purpose of the request; and delivery date and time. Write the following in the patient’s record or sheet authorized for such purpose: MLuq protocol Concluded.

## 4. Discussion

The response of all the professionals was negative, which gives at least internal validity to said protocol. However, the absence of literature makes us think that the protocol will also be useful in other geographies.

Unprecedentedly, this article presents a performance protocol for healthcare workers in emergency situations assisting victims of white weapon aggressions with a dual innovation, i.e., buth healthcare management-related and legal. On the one hand, the health care provided to the patient can generate weapon displacement leading to the rupture of large blood vessels, which will result in severe bleeding, pulmonary conditions, irreversible injuries, multiple organ dysfunction, and death [17]. However, the healthcare workers’ current performance is circumscribed to the existing resources and to the criterion of the assisting professional, with no protocols published for such an end—at least, according to the systematic review conducted in the first phase of this study.

The protocol has been designed with sanitary techniques and materials that are familiar to professionals in extra-hospital emergency teams in our healthcare setting. Both doctors and nurses could carry out all the procedures (except for the suture) to immobilize the weapon that would, at this stage, be in charge of the medical staff. Certainly, it is recommended that healthcare workers acquire skills to avoid contamination of biological traces because nowadays, this concern is usually not considered. In this sense, continuous training courses are currently being designed in our hospital where this protocol will shortly be implemented.

The proposed suture technique (purse string suture), although it is widely known in other scopes, has never been proposed as a technique to immobilize weapons inside the victims’ body in order to avoid the aforementioned complications during the assistance provided and subsequent transportation to the reference health center. The idea has been discussed and validated by the authors, who work in the surgical and emergency health areas, and who reached a consensus about its possible usefulness based on the efficacy shown in similar scenarios. Specifically, the idea emerged from its application in cardiovascular surgery—specifically, during cannulation of large blood vessels (auricles and ventricles)—to establish cardiopulmonary shunt. The technique stabilizes insertion of a cannula in the bed of a given anatomical area, keeping it immobile during the surgery and fixing it with greater or lesser pressure by using tourniquets or thread guides.

Regarding the type of swab and conservation used in the protocol, they are nylon swabs for sample collection. To date, traditional swabs are made of cotton fibers. In recent years, new swabs have been developed with short nylon fibers that are arranged perpendicularly. These new swabs have better performance than the traditional ones because they are more efficient in collecting evidence since the sample adheres to its surface instead of being absorbed. This further facilitates their conservation, since they can be kept at room temperature without the need for refrigeration, and improves their treatment in the laboratory, since the fibers make the elution process more efficient.

On the other hand, from the legal and forensic medicine point of view, this protocol also makes an important contribution since, as already commented, healthcare workers lacked indications that allowed them to perform their duties without interfering (or doing so minimally) in the chain of custody of biological traces that might later be used to identify the aggressor [18] and, consequently, as proofs of legal interest.

Regarding the number and modality of the photographs, it should first of all be noted that the suitability of recording images as a descriptive method of lesions is evident, although it must always be taken into account that the capture of images must always be panoramic (if they are made for location purposes and show the appearance of the place as it was found, it is convenient to take at least four shots at different angles) and general plans (they will serve to locate and relate evidence in the scene; if there are victims, these images must show how the position of the subject is related to the knife, at least two shots being convenient). It is true that the forensic method establishes two-dimensional (2D) photographs together with three-dimensional (3D) models and data and photogrammetry that are generally viewed on a computer screen, but it should be noted that these photographs attend to deceased victims, so they require a greater number of photographs in order to trace distances from crime scenes [19]. Therefore, for our MLuq protocol, photographs with general plans and panoramas of the scene are sufficient. In addition, photographs should not be used to interpret subtle or non-specific findings and can never be used to reliably diagnose lesions which has not been directly observed by examiners [20].

As a limitation, we can state that to the present day, the protocol is only conceptual, as the design phase has been concluded, but it has not yet been implemented. The incorporation of new maneuvers aimed exclusively at obtaining or maintaining biological samples for forensic or legal purposes could be poorly received by health personnel, alleging a greater workload in situations where it is essential to focus on the patient’s life. However, no healthcare worker consulted mentioned the increase in new maneuvers as a problem, so we anticipate that it will not be a problem. Additionally, we think that after the practical training that is currently being designed in our hospital prior to the implementation of the protocol has been integrated, it will allow for effective management of the situation, nullifying this perception should it occur to the healthcare workers.

Thus, there is counseling by renown professionals who have validated it from the technical point of view and ratified the absence of similar useful interventions in their everyday professional practice, thus granting qualitative weight to the protocol from a practical point of view, which should be further ratified with its implementation and evaluation through a follow-up study that is currently in the planning phase.

The refusal to recognize previous similar protocols by the experts showed the local validity of the protocol, and the absence of scientific literature that describes similar protocols in other countries indicates its possible global utility, either as we present it, or with adaptations pertaining to each new point of implantation.

In fact, although this protocol has not yet been implemented, at the moment, it has been presented and received with interest by different regions of the country (Andalusia, the Valencian Community, and the Balearic Islands) and is currently in the evaluation stage, prior to its implementation. Likewise, we do not rule out the possibility that it will arouse interest in other countries.

To ensure the success of the protocol, it will be necessary to implement educative activities aimed at raising awareness among healthcare workers who put it into practice, emphasizing that maintaining the chain of custody is an ethical and professional duty that is a critical procedure with respect to the admissibility of the evidence in a court of justice [21].

## 5. Conclusions

The MLuq protocol has been agreed by consensus in a multidisciplinary manner and is the first one to propose purse string suture as a weapon immobilization technique, avoiding complications that compromise the patient’s life, as well as a set of actions designed to obtain biological traces of legal interest and to preserve the chain of custody.

Thus, this protocol conceptually and procedurally equips healthcare workers who assist victims of stab wounds with knowledge that will allow them to be more effective in resolving these extra-hospital emergency situations. In post-mortem situations, it is not necessary to move the patient, and it is easier to remove, with more time and care, the possible traces of DNA that the attacker has left on the weapon. However, if the patient is alive, the priority is his/her stabilization and transfer to a specialized center, initiating maneuvers that contaminate or deteriorate the DNA template. This protocol informs healthcare professionals of the necessary procedures to minimize these problems, which frequently hinder or prevent the subsequent genetic identification of the aggressor, leading to the dismissal of evidence in a future trial. Finally, we can say that this protocol facilitates sanitary, forensic, and legal work around a stabbing case.

## 6. Patents

This protocol was registered in the Andalusian Territorial Registry of Intellectual Property with file number RTA-582-22.

## Data Availability

Not applicable.

## Supplementary Materials

The following supporting information can be downloaded at: https://www.mdpi.com/article/10.3390/healthcare11111573/s1, Material S1: SEARCH STRATEGIES; Material S2: RESULTS; Figure S1. PRISMA 2009 Flow Diagram.
