# Supplementary material for: MLuq Protocol: A Proposal for the Immobilization of the White Weapon, Preservation of DNA Traces, and Its Chain of Custody
Source: Healthcare (Basel). 2023 May 27;11(11):1573. doi: 10.3390/healthcare11111573 (PMC10253184; doi:10.3390/healthcare11111573)
Supplement: Supplementary file 1 [file healthcare-11-01573-s001.zip › healthcare-2244193-supplementary.pdf]

SUPLEMENTARY MATERIAL

SEARCH STRATEGIES
#1 (white weapon OR blade weapon OR stab*) AND (DNA preservation)
#2 DNA preservation protocol AND weapon
#3 DNA preservation protocol AND victim
#4 weapon immobilization AND stab*
#5 weapon immobilization protocol

RESULTS

<u>PUBMED</u>
#1 => 0 results
#2 => 18 results => no eligible results after reading title/abstract
#3 => 6 results => 0 no eligible results after reading title/abstract
#4 => 35 results => no eligible results after reading title/abstract
#5 => 8 results => no eligible results after reading title/abstract

SCOPUS
#1 and #4 => 0 results
#2 =>1 result => no eligible results after reading title/abstract
#3 => 6 results (4 duplicated in pubmed) => no eligible results after reading title/abstract
#5 => 3 results => no eligible results after reading title/abstract

EMBASE
#1, #2, #4 => 0 results
#3 => 3 results (1 duplicated in Pubmed and Scopus) => no eligible results after reading title/abstract
#5 => 3 results => no eligible results after reading title/abstract

TRIP DATABSE
#1 => 43 results => no eligible results after reading title/abstract
#2 => 36 results, no eligible results after reading title/abstract
#3 => 74 results (36 duplicated in #2) => no eligible results after reading title/abstract
#4 => 0 results
#5 => 34 results (34 duplicated in #2 y #3)

COCHRANE DATABASE AND LILACs
0 results for all strategies

SCIELO
#1 2 results => no eligible results after reading title/abstract
#2 #3 #4 #5 => 0 results

From: Moher D, Liberati A, Tetzlaff J, Altman DG, The PRISMA Group (2009). *P*referred *R*eporting *I*tems for *S*ystematic Reviews and *M*eta-*A*nalyses: The PRISMA Statement. PLoS Med 6(6): e1000097. doi:10.1371/journal.pmed1000097

For more information, visit www.prisma-statement.org.

Figure S1. RISMA 2009 Flow Diagram.

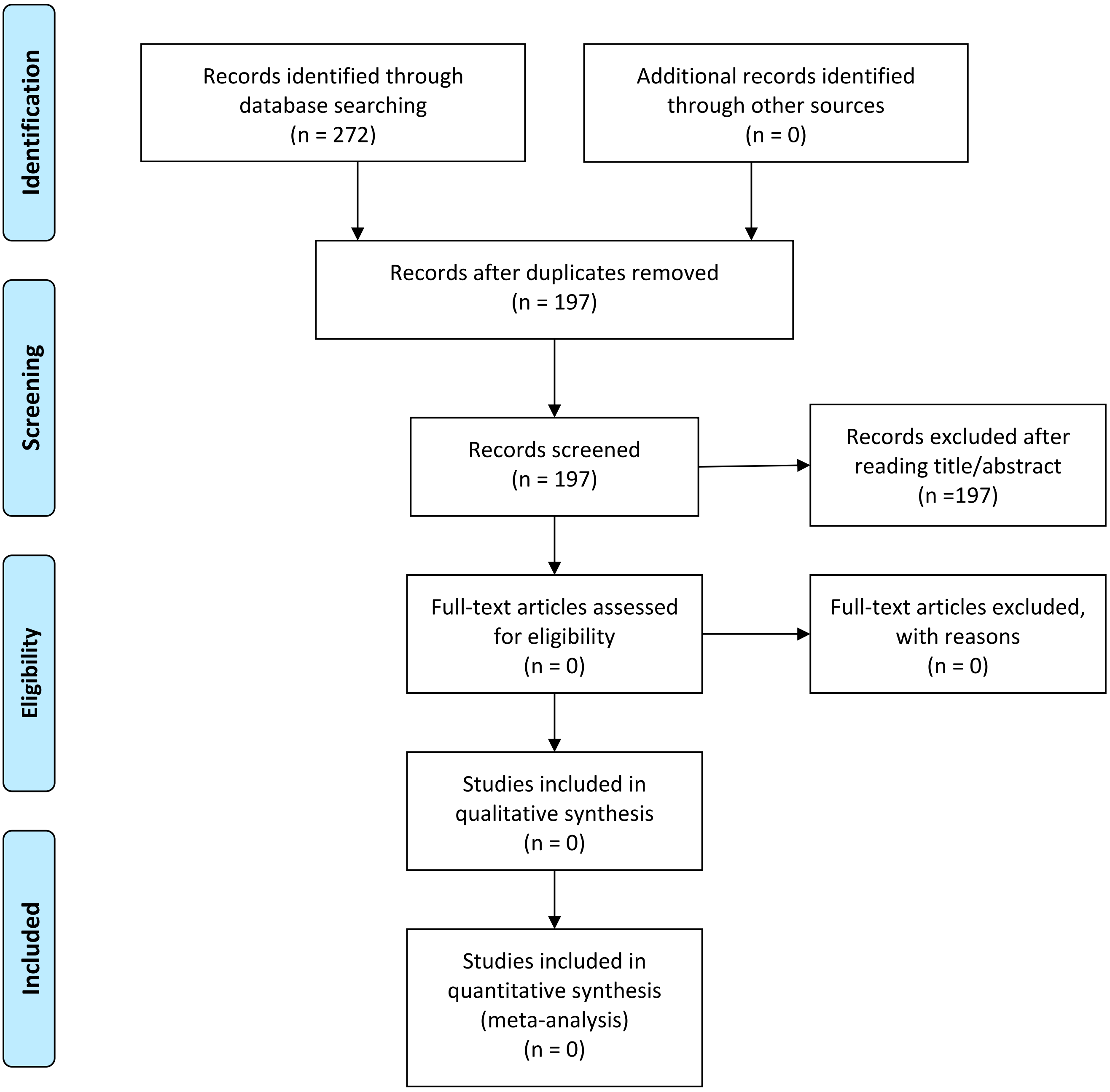

From: Moher D, Liberati A, Tetzlaff J, Altman DG, The PRISMA Group (2009). *P*referred *R*eporting *I*tems for *S*ystematic Reviews and *M*eta-*A*nalyses: The PRISMA Statement. PLoS Med 6(6): e1000097. doi:10.1371/journal.pmed1000097

For more information, visit www.prisma-statement.org.
